# The relationship between body mass index and health-related quality of life in COPD: real-world evidence based on claims and survey data

**DOI:** 10.1186/s12931-020-01556-0

**Published:** 2020-11-03

**Authors:** Manuel B. Huber, Christoph Kurz, Florian Kirsch, Larissa Schwarzkopf, Anja Schramm, Reiner Leidl

**Affiliations:** 1 Institute of Health Economics and Health Care Management, Helmholtz Zentrum München, Ingolstädter Landstraße 1, 85764 Neuherberg, Germany; 2 Munich School of Management and Munich Center of Health Sciences, Ludwig-Maximilians-Universität, Munich, Germany; 3 Comprehensive Pneumology Center Munich (CPC-M), Member of the German Center for Lung Research (DZL), Neuherberg, Germany; 4 IFT-Institute Fuer Therapieforschung, Working Group Therapy and Health Services Research, Munich, Germany; 5 AOK Bayern, Service Center of Health Care Management, Regensburg, Germany

**Keywords:** COPD, BMI, Health-related quality of life, Obesity, Real-world evidence

## Abstract

**Background:**

Body mass index (BMI) is an important parameter associated with mortality and health-related quality of life (HRQoL) in chronic obstructive pulmonary disease (COPD). However, informed guidance on stratified weight recommendations for COPD is still lacking. This study aims to determine the association between BMI and HRQoL across different severity grades of COPD to support patient management.

**Methods:**

We use conjunct analysis of claims and survey data based on a German COPD disease management program from 2016 to 2017. The EQ-5D-5L visual analog scale (VAS) and COPD Assessment Test (CAT) are used to measure generic and disease-specific HRQoL. Generalized additive models with smooth functions are implemented to evaluate the relationship between BMI and HRQoL, stratified by COPD severity.

**Results:**

11,577 patients were included in this study. Mean age was 69.4 years and 59% of patients were male. In GOLD grades 1–3, patients with BMI of around 25 had the best generic and disease-specific HRQoL, whereas in GOLD grade 4, obese patients had the best HRQoL using both instruments when controlled for several variables including smoking status, income, COPD severity, comorbidities, emphysema, corticosteroid use, and days spent in hospital.

**Conclusion:**

This real-world analysis shows the non-linear relationship between BMI and HRQoL in COPD. HRQoL of obese patients with mild to severe COPD might improve following weight reduction. For very severe COPD, a negative association of obesity and HRQoL could not be confirmed. The results hint at the need to stratify COPD patients by disease stage for optimal BMI management.

## Background

Despite being half as common as the most prevalent lung disease, asthma, chronic obstructive pulmonary disease (COPD) is associated with eight times higher mortality [1] and affects around 13.1% of humans worldwide [2]. COPD is associated with reduced health-related quality of life (HRQoL) [3]. Interestingly, the increasing problem of overweight [4] is associated with increased survival for COPD patients compared with their underweight and/or normal weight peers [5]. This association has been confirmed for a variety of other diseases including heart disease [6] and type 2 diabetes [7]. Some studies even extend this finding to the general population [8] and others refute it [9–11]. Although most related studies evaluated survival, HRQoL is an alternative and relevant outcome. In contrast to hard outcomes such as mortality, HRQoL resembles a multidimensional construct to measure perceived patient health status, which is important to improve patient management. Moreover, in contrast to single events such as mortality, HRQoL can be measured over time and allows the tracking of health status changes. HRQoL is also associated with body mass index (BMI) [12–16] and overweight is often manageable. Intervention options range from lifestyle changes to pharmacologic treatment to surgery [17]. Weight loss is achievable in most cases, and normalizing BMI can lead to remarkable improvement in health states [18]. Improving HRQoL is a key aim in German disease management guidelines for COPD patients [19], but overall prediction of COPD outcomes still remains difficult [20]. Despite being an important management variable, actual guidelines often fail to account for high BMI [21, 22] or cover obesity rather casually [23]. By evaluating the association of BMI and HRQoL across different COPD severity grades, this study aims to provide current easy-to-understand guidance for optimal weight recommendations in COPD patients.

## Methods

### Data

Claims and survey data were evaluated for this study. Claims data were acquired through AOK Bayern, the largest statutory health insurance in Bavaria with a market share of around 40%. Only patients who participated in the COPD disease management program (DMP) in the respective year—a structured treatment/surveillance program for specific chronic diseases—were included in this evaluation. Surveys were sent to all COPD DMP patients of AOK Bayern, when respective claims data were available for the year before the survey. The questionnaire included questions regarding disease-specific as well as generic HRQoL and COPD status (severity, exacerbations). Overall, the survey was sent to 49,664 patients on November 13, 2017. Of these patients, 14,753 (29.7%) responded. Claims and survey data were linked in a next step. Claims data included additional variables such as costs, health care service utilization, and medical parameters (e.g., forced expiratory volume in the first second (FEV1)). When several observations were available for the same variable in the year before the survey, only the latest measurement was included. Patients without HRQoL or FEV1 measurement or implausible values were excluded. Following this filtering, 11,577 patients remained for the evaluation. The ethics committee of the Ludwig Maximilians University, Munich, approved the study (vote no. 17-358). Participants provided written informed consent at the time of inclusion in the DMP.

### Disease severity

Disease severity was assessed via the GOLD COPD staging system [24]. GOLD grades are based on the percentage of predicted forced FEV1 and require one post-bronchodilator diagnosis of COPD. The four GOLD severity grades are defined as 1 = mild (> 80% of FEV1 predicted), 2 = moderate (50–79% predicted), 3 = severe (30–49% predicted), and 4 = very severe (< 30% predicted).

### Measures

BMI was calculated by dividing patient weight in kg by height in meters squared. HRQoL instruments transform health states into numerical scores and can be used to evaluate and track the actual health perception of patients [25]. To measure the generic and disease-specific HRQoL, the EQ-5D-5L [26] and the COPD Assessment Test (CAT) [27] were used. The EQ-5D-5L is a widely accepted and descriptive generic instrument. It consists of five dimensions (mobility, self-care, usual activity, pain/discomfort, anxiety/depression) and a visual analog scale (VAS). The VAS is a continuous response scale ranging from 0 (worst state) to 100 (best state). Responders rate their current health state by selecting a point on this scale. The 5L version of the EQ-5D includes five answer levels for each of the five dimensions, ranging from no problems [1] to extreme problems [5]. VAS results are a more general reflection of health perception, whereas the descriptive dimensions are directed at specific aspects of health. Psychometric properties of the EQ-5D-5L have been validated [26].

The CAT is a disease-specific instrument. It consists of eight dimensions with six answer levels (0 = best to 5 = worst). Thus, the worst sum score for the CAT is 48 and the best is 0. The CAT includes several disease dimensions of COPD including but not limited to cough, mucus production, sleep, and energy.

### Model

We use a generalized additive model (GAM) [28] to evaluate the association between BMI and HRQoL, stratified by GOLD grade. GAMs have the advantage of being able to portray a non-linear relationship between variables, which is likely for the association between BMI and HRQoL in COPD, as underweight and overweight are connected to detriments. Furthermore, they are easy to interpret. The applied model uses a backfitting algorithm to fit the weighted additive models iteratively. The model uses spline-based smoothing to evaluate the influence of BMI. The included co-variables for this model are age, gender, BMI, income, smoking status, Elixhauser Comorbidity Index [29], days spent in hospital, exacerbations (medium, severe), FEV1 percent predicted, daily defined doses of corticosteroids, and emphysema. To determine the number of medium or severe exacerbations, patients were asked whether, during the last 12 months, their COPD symptoms (e.g., increased dyspnea, mucus production) worsened significantly and required a hospital stay (severe exacerbation) or doctor visit (medium exacerbation). A summary of all model variables and their respective sources can be found in Additional file: Table S1. To address possible multicollinearity issues, we calculated the variance inflation factor. It was below any meaningful threshold, indicating no problems with multicollinearity. An unadjusted model was calculated for sensitivity analysis. We calculated the 95% confidence intervals using maximum likelihood estimation. All evaluations were conducted with R [30].

## Results

A total of 11,577 complete cases were included in the evaluation (Table 1). Of these, 59% were male, mean age was around 69.4 years, and more than 81.8% were defined as never smokers during DMP, meaning that they did not smoke during participation in the DMP (the last 1–10 years). The majority of patients were in GOLD grade 2 (5495). Only around 746 patients were in GOLD grade 4. Mean VAS score was 55.6 and mean CAT score was 19.9. Hospital days, number of exacerbations, emphysema, and corticosteroid use increased with increasing GOLD grade, whereas health perception declined.

**Table 1:** Patient characteristics

	GOLD 1 (N = 2,534)	GOLD 2 (N = 5,495)	GOLD 3 (N = 2,802)	GOLD 4 (N = 746)	Total (N = 11,577)
Age (years)	69.6 (10.7)	69.6 (10.2)	69.6 (9.4)	66.8 (8.7)	69.4 (10.0)
Gender					
Female	1,200 (47.4%)	2,300 (41.9%)	1,050 (37.5%)	201 (26.9%)	4,751 (41.0%)
Male	1,334 (52.6%)	3,195 (58.1%)	1,752 (62.5%)	545 (73.1%)	6,826 (59.0%)
BMI	29.5 (5.6)	29.5 (6.0)	28.1 (6.1)	26.3 (5.9)	28.9 (6.0)
Smoking status^a^					
Never smoker during DMP	2,207 (87.1%)	4,561 (83.0%)	2,192 (78.2%)	515 (69.0%)	9,475 (81.8%)
Active	156 (6.2%)	433 (7.9%)	290 (10.3%)	100 (13.4%)	979 (8.5%)
Ex-smoker	171 (6.7%)	501 (9.1%)	320 (11.4%)	131 (17.6%)	1,123 (9.7%)
Income (EUR)	13,220.4 (12,181.7)	12,723.4 (11,942.6)	13,903.2 (11,1739.9)	11,230.4 (10,924.2)	13,021.6 (55,941.3)
Elixhauser index	5.3 (2.9)	5.5 (3.0)	5.4 (3.0)	5.1 (2.9)	5.4 (3.0)
Hospital days	5.4 (15.8)	5.9 (14.9)	8.1 (17.4)	10.2 (17.7)	6.6 (16.0)
FEV1% predicted	89.6 (6.0)	65.0 (8.4)	41.0 (5.6)	23.8 (4.8)	61.9 (20.6)
Medium exacerbations	0.5 (1.8)	0.6 (1.8)	0.8 (2.0)	1.0 (2.1)	0.7 (1.9)
Severe exacerbations	0.1 (0.8)	0.2 (1.1)	0.4 (1.5)	0.8 (2.0)	0.3 (1.2)
Emphysema					
No	2,297 (90.6%)	4,738 (86.2%)	2,052 (73.2%)	426 (57.1%)	9,513 (82.2%)
Yes	237 (9.4%)	757 (13.8%)	750 (26.8%)	320 (42.9%)	2,064 (17.8%)
DDD of corticosteroids	20.9 (70.2)	27.1 (82.5)	57.3 (124.7)	98.8 (170.6)	37.7 (101.9)
EQ-5D-5L VAS	58.3 (20.9)	57.6 (20.8)	52.0 (20.8)	44.7 (20.4)	55.6 (21.1)
CAT	18.5 (8.3)	19.3 (8.3)	21.6 (8.4)	23.6 (8.4)	19.9 (8.4)

Percentage or standard deviation in brackets

^a^Smoking status could only be assessed for the time span beginning at patient DMP entry, but no longer than for the last 10 years

Additional file 1: Table S2 illustrates patient population characteristics by BMI group. Around 2% of patients were underweight, 25% normal weight, 36% overweight (BMI ranging from 25 to 29.9), and 37.5% obese (BMI ≥ 30). Underweight is also associated with more hospital days and higher GOLD grade. Normal weight patients had the highest average income. There was not much difference in depression/anxiety scores (fifth dimension of the EQ-5D-5L), but there was a strong negative correlation between BMI and emphysema. Underweight patients had the highest levels of emphysema and obese patients had the lowest rate.

**Fig. 1:**
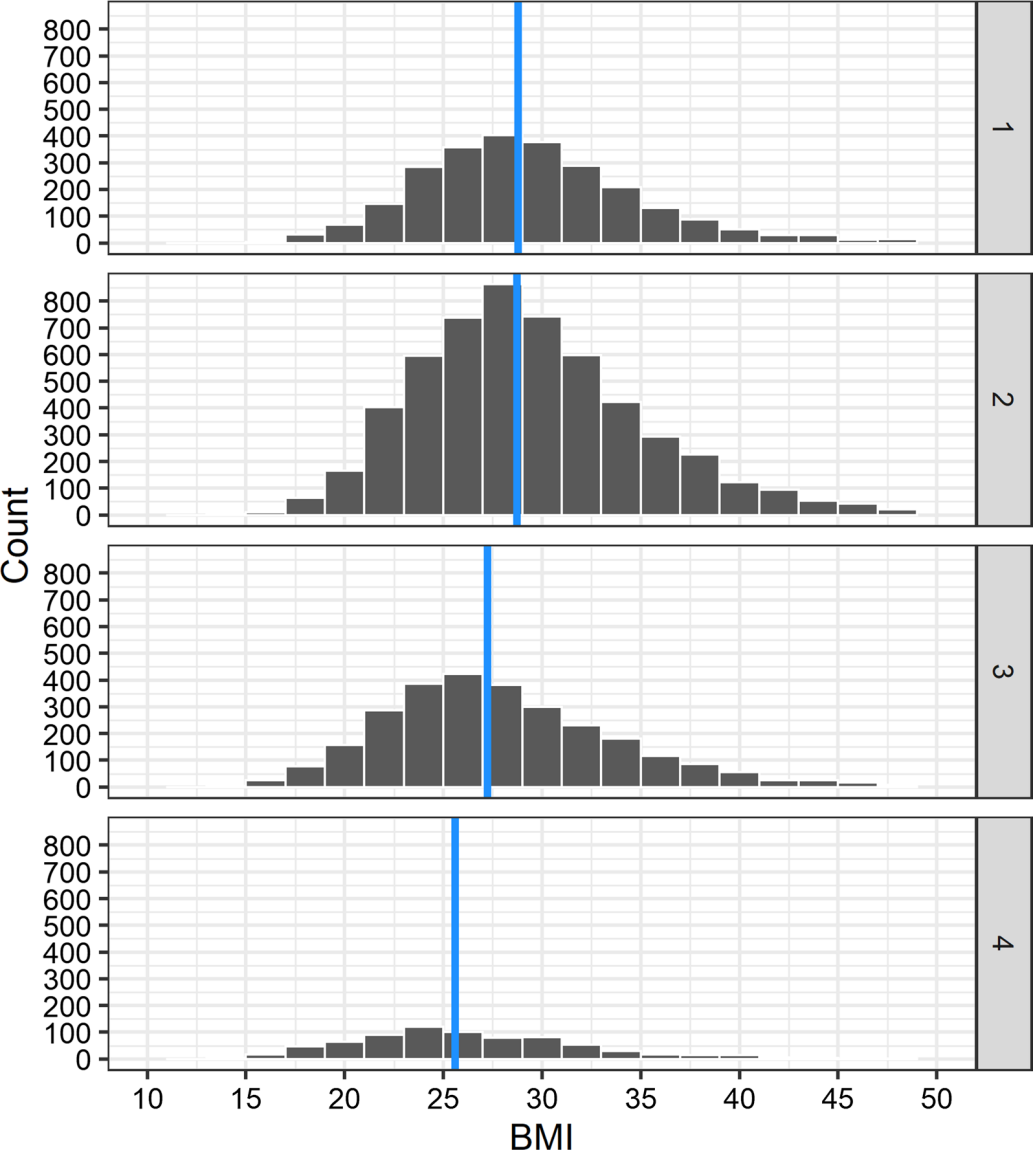
Histograms for BMI frequency, stratified by GOLD grade. Note: GOLD grades displayed on right side, count refers to the number of patients with the depicted BMI in the respective group

BMI was normally distributed across GOLD grades 1–3 (Fig. 1) but looked uniform/bimodal for GOLD 4. Overall, the median BMI declined slightly across GOLD grades from 28.7 in GOLD 1 to 25.7 in GOLD 4. The percentage of patients with BMI ≥ 40 (extremely obese) in each grade was 4.41 (GOLD 1), 5.29 (GOLD 2), 4.13 (GOLD 3), and 2.81 (GOLD 4).

**Fig. 2:**
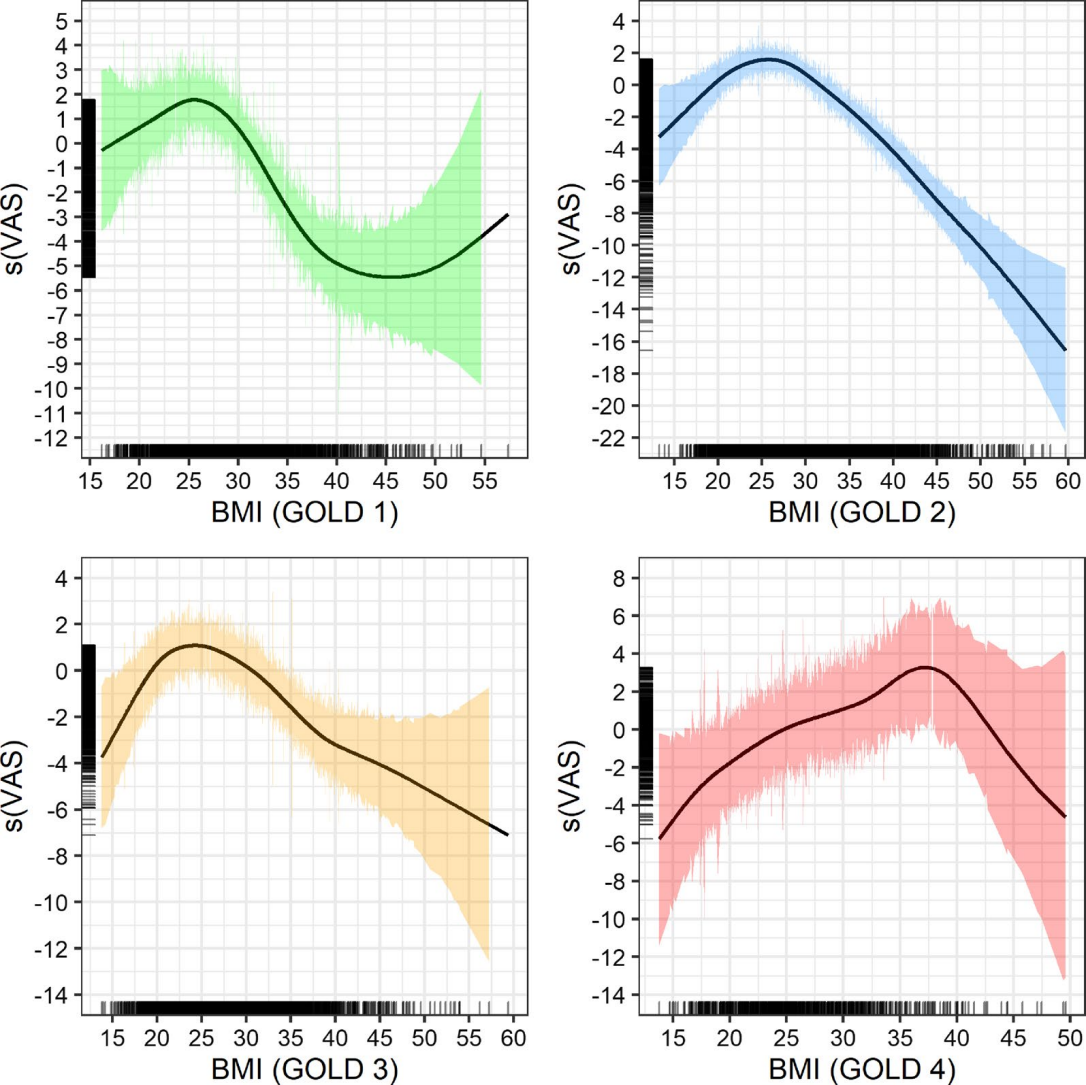
GAM plots for BMI and VAS, stratified by GOLD class. Note: Smoothed model output for VAS on y-axis. Rug plots (black bars) to the left and right indicate the number of observations per BMI measure

Figure 2 depicts four GAM plots illustrating the association between BMI and VAS, stratified by GOLD grade. In GOLD grades 1–3, a BMI of around was 25 associated with the highest HRQoL, whereas in GOLD grade 4, a BMI of around 37 was associated with the best health perception. Based on the reduced number of observations starting at a BMI of 40, confidence intervals widened for GOLD grades 1, 3, and 4 but not so much for GOLD 2, where most observations were available. In GOLD grade 4, obesity was associated with higher HRQoL. Underweight was associated with reduced HRQoL in every GOLD grade. The optimal BMI range for high HRQoL was between 25 and 30 for GOLD grades 1 and 2, between 20 and 30 for GOLD grade 3, and between 25 and 43 for GOLD 4 (confidence intervals widen significantly after this point).

**Fig. 3:**
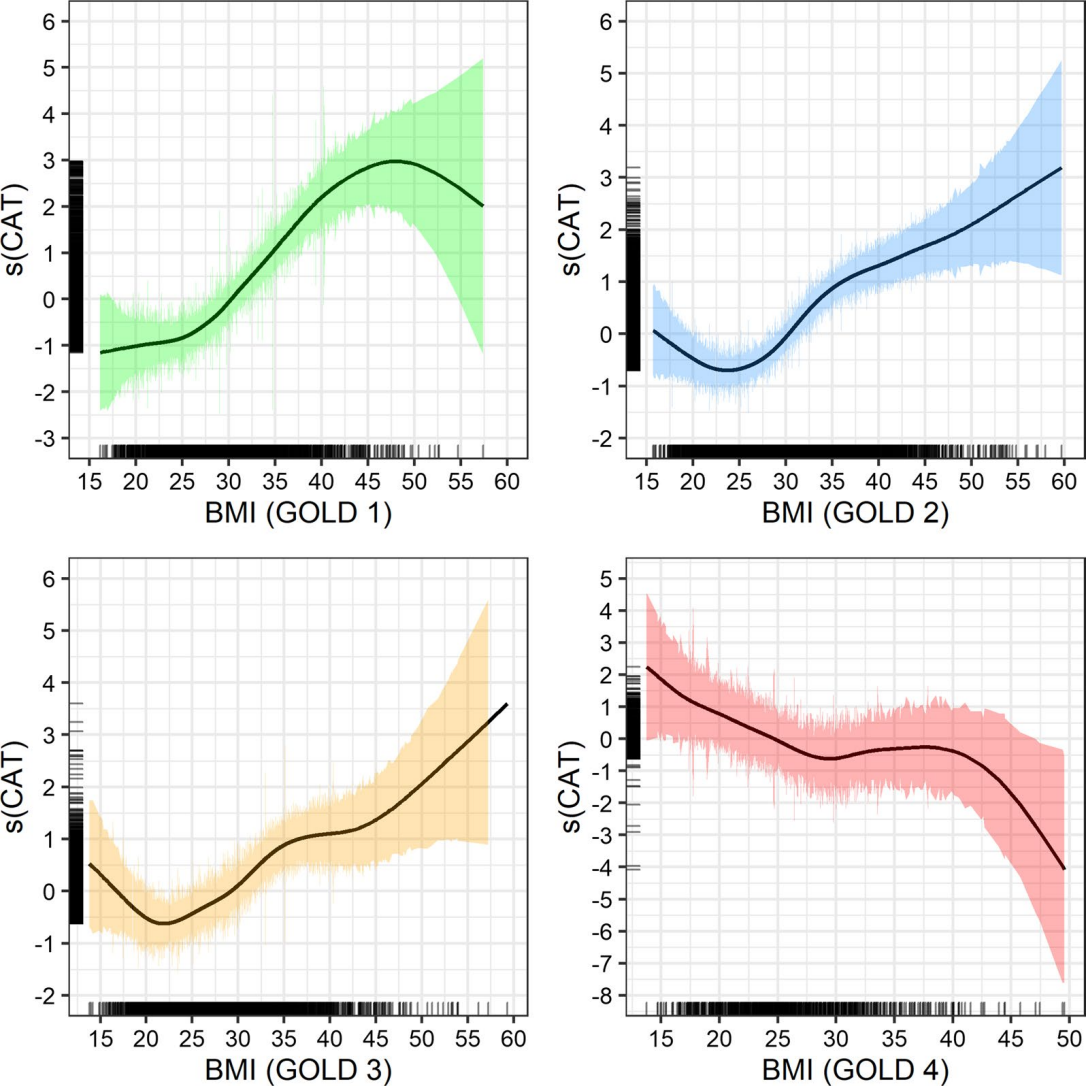
GAM plots for BMI and CAT, stratified by GOLD class. Note: Smoothed model output for CAT on y-axis. Rug plots (black bars) to the left and right indicate the number of observations per BMI measure

In their direction, the CAT-based results are comparable to the VAS-based findings (Fig. 3). Lower CAT scores indicate better disease-specific HRQoL and, for GOLD grades 1–3, patients with normal weight reported the best health perception. However, comparable to generic HRQoL, obese patients in GOLD grade 4 reported better HRQoL than their normal weight peers.

**Fig. 4:**
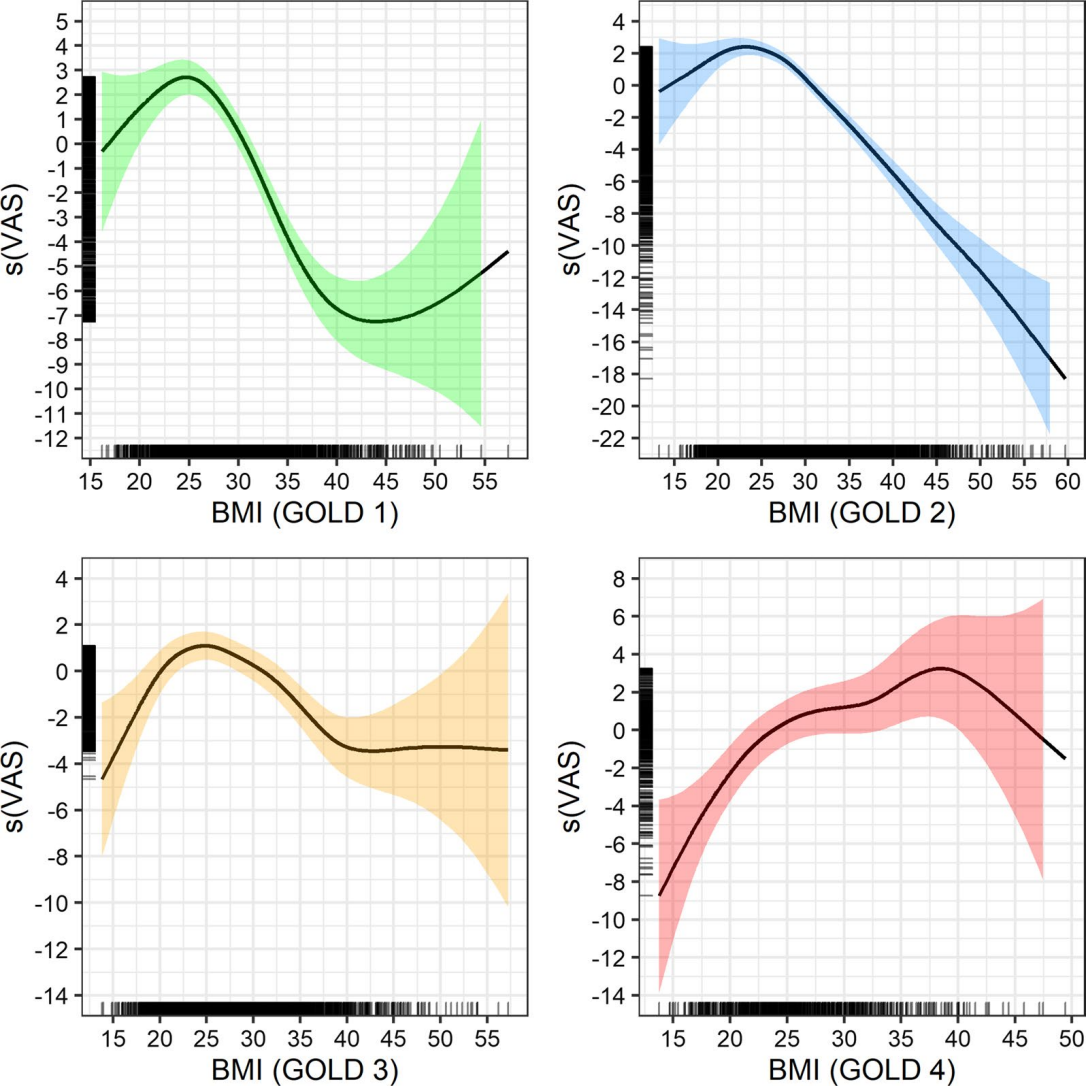
Unadjusted GAM plots for BMI and VAS, stratified by GOLD class. Note: Smoothed model output for VAS on y-axis. Rug plots (black bars) to the left and right indicate the number of observations per BMI measure

For sensitivity analysis, the unadjusted model, only including BMI and VAS, delivered comparable results to the adjusted models (Fig. 4).

## Discussion

Our results illustrate that normal weight in COPD is associated with increased HRQoL among DMP participants in GOLD grades 1–3, but obese participants in grade 4 have higher HRQoL than their normal weight peers. The findings are robust for several control variables including sociodemographic data and disease status. Moreover, we confirmed findings from other studies [31, 32] that reduced BMI is associated with high emphysema rates. A recent review by Sun et al. [33] indicates that reduced BMI is also associated with increased lung function decline. Consequently, patients with low or normal weight in GOLD 4 may be on a worse disease trajectory than their higher BMI counterparts who have slower lung function decline. Moreover, GOLD grade 4 resembles a special case as it is the last severity stage and patients are close to running out of reserves. Based on the observational nature of this study and the available variables, more advanced clinical research is needed to identify the actual causes of the shifted association between BMI and HRQoL in GOLD grade 4. Additional confounding and colliding also have to be accounted for.

### Potential confounders

Regarding the impact of BMI on mortality, there is a rich debate on confounders in the context of the obesity paradox in COPD. For potential confounders in our study, we refer to this debate. Spelta et al. [34] name several possible explanations for the obesity paradox: higher BMI may be associated with physiological changes that restrict but do not obstruct lung function and, hence, lead to worse GOLD classification for higher weight patients. In these cases, obesity and not COPD is the driver behind lower FEV1. Figure 1 illustrates the median BMI of the study population, stratified by GOLD severity. In our study, median BMI declined with severity grade, especially between GOLD grades 2 and 4. Mean BMI (not shown here) also declined with increasing GOLD grade. This indicates that there is no substantial correlation between higher weight and reduced lung function in our study population.

Spelta et al. 2018 also refer to evidence that higher BMI is associated with less hyperinflation. Owing to lack of respective data, it is not possible to evaluate the distribution of hyperinflation among our study population. Yet other studies show that, although hyperinflation increases mortality risk [35], only static but not the more common dynamic hyperinflation seems to be associated with reduced HRQoL [36].

Emphysema correlates with a loss of body fat as well as skeletal muscles [37]. Emphysema is also associated with reduced HRQoL in COPD patients [38] and is more prevalent among higher GOLD severity grades [39]. Thus, emphysema may explain lower HRQoL and lower weight among COPD patients with GOLD grade 4. However, we controlled for emphysema by including the respective ICD-10 code, and no significant influence on the overall findings was observed.

BMI has been criticized for not indicating body composition or body fat percentage [40]. Instruments that include waist adiposity, such as “A Body Shape Index” [41], may therefore be better predictors of mortality [42]. It was not possible to account for this issue, but it seems unlikely that patients with severe airway restriction (GOLD 4) have better body composition than their controls in lower severity grades.

Smoking is also a potential confounder and may lead to reverse causation [43], as it reduces hunger/calorie intake and increases energy consumption. Like emphysema, smoking status was controlled for in this study, but no significant effect on results was observed.

Another potential confounder is increased surveillance and better care for obese patients. They may receive more intense care and may stay in hospital for longer periods of time [8]. Thus, hospital days in the year before the questionnaire were controlled for, but the findings did not change.

Income is an indicator of socioeconomic status. Patients with lower income may also have lower HRQoL and higher BMI. So, income was accounted for.

Another issue, collider bias, is a form of selection bias, where exposure and outcome cause a third variable [44]. Colliders should be removed from models, as they distort estimations. Some studies state that collider bias is responsible for and invalidates the obesity paradox [45]. An analysis of why collider bias seems to be no valid explanation for our findings can be found in the Additional file 1 (see section on collider bias).

In sum, this study has been able to control for most of the important confounders of BMI impact discussed in the context of COPD, or at least hints have been found that potential bias of factors not controlled or controlled for (collider bias) might be low.

### Related research

Previous studies have indicated an association between underweight and higher mortality in COPD [46, 47]. This corresponds with our finding that underweight patients have more hospital days, more exacerbations, and decreased lung function. Another study evaluated data from a double-blind randomized controlled trial carried out in 1368 centers in 43 countries and concluded that BMI over 40, in patients with moderate COPD and increased cardiovascular risk, does not reduce mortality, whereas less severely obese patients (BMI 30–35) show a clear survival benefit compared with BMI 20–25 [48]. This somewhat resembles our study finding where BMI from 35 to 40 was associated with higher HRQoL, whereas BMI scores > 40 were associated with HRQoL declines in GOLD 4 patients. A study by Landbo et al. [5] also found that COPD grade had a significant influence on the association of BMI and mortality. For less severe COPD, normal weight appears to be optimal, but for severe COPD, overweight appears to be beneficial [5]. A narrative review by Spelta et al. [34] evaluated the nine most important studies on the obesity paradox and concluded that the “protective” effect of obesity is most evident for the severe grades of COPD but not for mild to medium airway restriction. Our study findings also point in this direction. The stratification by severity grade could explain why previous, unstratified evaluations [49, 50] reported conflicting results on the association between obesity and HRQoL in COPD.

### Clinical relevance

Minimal important differences (MIDs) define which changes in HRQoL are meaningful for patients. For example, the MID for EQ-5D-5L VAS in COPD was estimated to be around 6.9 in a study from 2016 [26]. To achieve this MID, patients in GOLD stage 2 would need to lose around 14 BMI points. A reduction of this magnitude is difficult to achieve. Although intervention options can be ordered systematically from lifestyle changes to pharmacotherapy to surgery [17], only the latter seems to be effective enough in achieving comparable long-term BMI reductions [51] with acceptable cost-effectiveness [52], when patients are not willing to rigorously change their lifestyle. Surgery has also been found to lower COPD impact [53]. Only doctors and patients can make the respective decisions, and they should always account for possible side effects or unforeseen consequences. However, to avoid the need for surgical intervention or rigorous lifestyle changes, prevention of obesity would certainly be preferable. To improve prevention, COPD patients should be put under special surveillance either when they gain weight quickly or when they reach certain BMI thresholds. Another important but more critical aspect is the avoidance of reaching HRQoL decrements that are relevant to the patient. For example, in GOLD grade 2, patients above BMI 42 would need a HRQoL improvement > MID to reach the optimum HRQoL in their stratum. Reaching a BMI below 42 for patients above this threshold would take them out of the described risk group.

The advantage of obesity treatment, contrary to the treatment of other diseases, is the underlying principle. To lose weight, calorie input has to be smaller than calorie expenditure. Both factors can be directly influenced and lead to immediate improvement when accounted for. Many other diseases are based on more complex coherencies, where cause and effect have not been identified and available treatments are far less effective.

### Limitations and strengths of this study

One limitation of this study is the lack of adjustment for lung hyperinflation and body composition. However, as stated in the discussion section, the influence of lung hyperinflation is unlikely to change study outcomes significantly, as the more prevalent dynamic form apparently has little influence on HRQoL. BMI fails to reflect body composition and does not allow detection of the increased mortality risk associated with sarcopenia. Supplementary measures such as hip–waist ratio are needed to acquire a more comprehensive picture of the risks associated with patient weight. As only DMP participants from AOK Bayern were included in this study and the response rate was 30%, selection bias is likely. However, on account of the relatively large number of observations, the findings regarding the association of BMI and HRQoL should still be valid for broader populations of COPD patients. Moreover, as DMP participation is associated with increased quality of care [54], the gap in HRQoL could be even higher in a non-DMP setting. Based on study design, a more representative approach was not possible.

A clear strength of this study is the large number of observations and the linkage of claims and survey data that enabled the evaluation of HRQoL. In contrast to mortality, HRQoL allows the guiding of patient management along the course of chronic disease and represents no binary outcome. Other strengths of this study are the stratification by severity grade, the number of included control variables, and the sensitivity analysis including an unadjusted model. To our knowledge, this study is the first to evaluate the association between BMI, HRQoL, and COPD severity grade based on a large number of observations from claims as well as survey data. Although this study could not solve all the issues associated with the connection of BMI and HRQoL in COPD, it certainly provides much needed and differentiated evidence to improve patient management.

## Conclusion

Obese COPD DMP patients in GOLD grades 1–3 report lower HRQoL, whereas obese patients in GOLD grade 4 report higher HRQoL than their normal weight peers. This finding is robust for a wide variety of control variables including, but not limited to, smoking status, income, emphysema, days spent in hospital, and exacerbations. Based on these findings, body weight is significantly associated with patient health perception. Weight reduction remains an important strategy that may improve HRQoL of COPD patients in the mild to severe grades of COPD. However, for the very severe grade, obesity (BMI ranging from 30 to 40) apparently is not associated with a decrease of the patients’ subjective well-being and hence might not constitute a primary treatment goal unless medical needs require it. The actual causes of this seemingly protective effect still need to be identified.

## Supplementary information

**Additional file 1: Table S1.** Model variables and source. **Table S2.** Patient population by BMI group.

## Data Availability

The datasets generated and/or analyzed during the current study are not publicly available according to the data protection concept approved by the responsible data security officials and the ethics committee.
