# Supplementary material for: The relationship between body mass index and health-related quality of life in COPD: real-world evidence based on claims and survey data
Source: Respir Res. 2020 Nov 3;21:291. doi: 10.1186/s12931-020-01556-0 (PMC7607880; doi:10.1186/s12931-020-01556-0)
Supplement: Supplementary file 1 — Additional file 1: Table S1. Model variables and source. Table S2. Patient population by BMI group. [file 12931_2020_1556_MOESM1_ESM.docx]

| **Additional Table 1** Model variables and source | |
| --- | --- |
| **Stratification variable** | **Source** |
| GOLD Stage (1-4) | DMP documentation (estimated with the GLI Macro) |
| **Predictor variables** |  |
| Age | Claims data |
| Gender | Claims data |
| BMI | DMP documentation (weight an height) |
| Smoke status | DMP documentation |
| FEV1 percent predicted | DMP documentation |
| Severe exacerbations | Survey data (hospital visit due to exacerbation) |
| Medium exacerbations | Survey data (doctor visit due to exacerbation) |
| Daily defined doses of corticosteroids | Claims data |
| Hospital days irrespective of cause | Claims data |
| Income in the year before survey | Claims data |
| Elixhauser index | Claims data |
| Emphysema | Claims data |
| **Outcome variables** |  |
| EQ-5D-5L VAS | Survey data |
| CAT score | Survey data |
|  |  |

| **Additional Table 2** Patient population by BMI group | | | | | | | |
| --- | --- | --- | --- | --- | --- | --- | --- |
|  | **Underweight (N=195, 1,8%)** | **Normal (N=2705, 24,7%)** | **Overweight (N=3950, 36,0%)** | **Class1 (N=2550, 23,2%)** | **Class2 (N=1053, 9,6%)** | **Class3 (N=517, 4,7%)** | **Total (N=10970)** |
| **Age** | 66.9 (10.0) | 68.9 (10.4) | 70.4 (10.0) | 70.1 (9.7) | 67.8 (9.5) | 65.3 (9.8) | 69.4 (10.0) |
| **Gender** |  |  |  |  |  |  |  |
| F | 117 (60.0%) | 1273 (47.1%) | 1410 (35.7%) | 942 (36.9%) | 445 (42.3%) | 256 (49.5%) | 4443 (40.5%) |
| M | 78 (40.0%) | 1432 (52.9%) | 2540 (64.3%) | 1608 (63.1%) | 608 (57.7%) | 261 (50.5%) | 6527 (59.5%) |
| **BMI** | 17.3 (1.1) | 22.7 (1.7) | 27.5 (1.4) | 32.2 (1.4) | 37.1 (1.4) | 44.3 (3.8) | 28.9 (6.0) |
| **Smoking status** |  |  |  |  |  |  |  |
| Active smoker | 22 (11.3%) | 292 (10.8%) | 309 (7.8%) | 198 (7.8%) | 77 (7.3%) | 27 (5.2%) | 925 (8.4%) |
| Ex-smoker | 21 (10.8%) | 270 (10.0%) | 362 (9.2%) | 248 (9.7%) | 110 (10.4%) | 48 (9.3%) | 1059 (9.7%) |
| Never smoker | 152 (77.9%) | 2143 (79.2%) | 3279 (83.0%) | 2104 (82.5%) | 866 (82.2%) | 442 (85.5%) | 8986 (81.9%) |
| **Income** | 10469.7 (10806.8) | 14727.6 (113854.3) | 13074.4 (12057.5) | 12514.4 (11232.6) | 12233.5 (10808.2) | 11863.3 (11088.8) | 13167.8 (57421.3) |
| **Elixhauser index** | 4.4 (2.5) | 4.5 (2.7) | 5.1 (2.8) | 6.0 (3.0) | 6.8 (3.0) | 6.9 (3.0) | 5.4 (3.0) |
| **Hospital days** | 10.8 (22.0) | 6.9 (15.3) | 6.0 (14.6) | 6.6 (18.5) | 6.9 (16.0) | 7.2 (16.5) | 6.6 (16.1) |
| **FEV1% predicted** | 46.0 (21.7) | 57.5 (21.3) | 63.2 (20.3) | 64.7 (19.8) | 64.5 (18.8) | 63.7 (18.4) | 62.0 (20.5) |
| **GOLD stage** |  |  |  |  |  |  |  |
| 1 | 22 (11.3%) | 481 (17.8%) | 916 (23.2%) | 648 (25.4%) | 232 (22.0%) | 112 (21.7%) | 2411 (22.0%) |
| 2 | 52 (26.7%) | 1133 (41.9%) | 1901 (48.1%) | 1274 (50.0%) | 564 (53.6%) | 274 (53.0%) | 5198 (47.4%) |
| 3 | 68 (34.9%) | 825 (30.5%) | 923 (23.4%) | 516 (20.2%) | 223 (21.2%) | 113 (21.9%) | 2668 (24.3%) |
| 4 | 53 (27.2%) | 266 (9.8%) | 210 (5.3%) | 112 (4.4%) | 34 (3.2%) | 18 (3.5%) | 693 (6.3%) |
| **Medium exacerbations** | 1.2 (2.3) | 0.6 (1.8) | 0.6 (1.9) | 0.6 (1.9) | 0.6 (1.8) | 0.9 (2.3) | 0.7 (1.9) |
| **Severe exacerbations** | 0.6 (1.7) | 0.3 (1.3) | 0.2 (1.1) | 0.3 (1.2) | 0.3 (1.2) | 0.2 (1.0) | 0.3 (1.2) |
| **Emphysema** |  |  |  |  |  |  |  |
| No | 110 (56.4%) | 1952 (72.2%) | 3289 (83.3%) | 2226 (87.3%) | 949 (90.1%) | 481 (93.0%) | 9007 (82.1%) |
| Yes | 85 (43.6%) | 753 (27.8%) | 661 (16.7%) | 324 (12.7%) | 104 (9.9%) | 36 (7.0%) | 1963 (17.9%) |
| **DDD of corticosteroids** | 84.3 (152.6) | 48.0 (119.4) | 33.8 (90.6) | 33.6 (94.6) | 31.2 (97.5) | 31.9 (103.1) | 37.8 (102.3) |
| **EQ_5DL_1** | 2.7 (1.1) | 2.3 (1.1) | 2.3 (1.1) | 2.6 (1.1) | 2.8 (1.1) | 2.9 (1.1) | 2.5 (1.1) |
| **EQ_5DL_2** | 2.1 (1.3) | 1.7 (1.1) | 1.6 (1.0) | 1.7 (1.0) | 1.9 (1.1) | 2.0 (1.2) | 1.7 (1.1) |
| **EQ_5DL_3** | 2.8 (1.2) | 2.3 (1.2) | 2.3 (1.1) | 2.4 (1.1) | 2.6 (1.1) | 2.7 (1.2) | 2.4 (1.1) |
| **EQ_5DL_4** | 2.7 (1.1) | 2.5 (1.0) | 2.5 (1.0) | 2.7 (1.0) | 2.8 (1.0) | 3.0 (1.1) | 2.6 (1.0) |
| **EQ_5DL_5** | 2.4 (1.2) | 2.0 (1.1) | 1.9 (1.0) | 2.0 (1.0) | 2.1 (1.1) | 2.2 (1.1) | 2.0 (1.1) |
| **EQ-5D-5L VAS** | 47.5 (21.9) | 56.7 (21.7) | 57.6 (20.9) | 55.5 (20.5) | 52.4 (20.5) | 50.6 (20.3) | 55.9 (21.1) |
| **CAT** | 22.7 (9.1) | 19.5 (8.5) | 19.2 (8.3) | 20.3 (8.1) | 21.2 (8.1) | 21.6 (8.3) | 19.9 (8.4) |
| **Problem score** | 12.8 (4.8) | 10.8 (4.5) | 10.6 (4.3) | 11.3 (4.4) | 12.1 (4.4) | 12.9 (4.6) | 11.1 (4.5) |
| Note: Corresponding BMI ranges from underweight to obesity class 3: < 18.5, 18.5 to 24.9, 25 to 29.9, 30 to 34.9, 35 to 39.9, greater than 40. | | | | | | | |

**Collider bias**

Collider bias is a form of selection bias, where exposure and outcome cause a third variable (1). Colliders should be removed from models, since they distort estimations. Some studies state that the collider bias is responsible for and invalidates the obesity paradox (2). However, research suggests that collider bias for the obesity paradox – mortality and not HRQoL were evaluated – is small compared with the causal relationships between the variables (3). While collider bias is often possible, it may not be plausible and other explanations need to be found (4). Moreover, based on our findings, it is questionable why collider bias would only reverse the association of BMI and HRQoL in GOLD severity grade 4 but not in grades 1 to 3. For sensitivity analysis, we also ran the unadjusted model that only included BMI as exposure and HRQoL as outcome (S1). This model suffers from possible confounding but does not control for possible colliders. The result did not change the overall findings in any relevant form. Proponents of collider bias recommend directed acyclic graphs (DAGs), a graphical representation of causal relationships between variables (5), as solution to identify colliders and reduce bias. However, there are several reasons that make DAGs extremely impractical for this study context. First of all, DAGs in literature are extremely simplistic when assigning causality to variables (6). The association between depression and obesity illustrates this issue. Does depression cause obesity (7) or does obesity cause depression (8) or is the association bi-directional (9)? Different causal relationships can exist for different patients. This is not only a source for significantly increased uncertainty but also for possible bias, since it becomes very difficult for scientists to find objective approaches to select and assign causal evidence that is not influenced by reductive and incomplete decision-making. Moreover, DAGs make no assumptions about the strength of respective associations and they allow creative proponents to identify possible collider bias in nearly any evaluation (6).

**Additional references**

1. Cole SR, Platt RW, Schisterman EF, Chu H, Westreich D, Richardson D, et al. Illustrating bias due to conditioning on a collider. Int J Epidemiol. 2010;39(2):417-20.

2. Banack HR, Kaufman JS. From bad to worse: collider stratification amplifies confounding bias in the “obesity paradox”. European Journal of Epidemiology. 2015;30(10):1111-4.

3. Sperrin M, Candlish J, Badrick E, Renehan A, Buchan I. Collider Bias Is Only a Partial Explanation for the Obesity Paradox. Epidemiology (Cambridge, Mass). 2016;27(4):525-30.

4. Glymour MM, Vittinghoff E. Commentary: selection bias as an explanation for the obesity paradox: just because it's possible doesn't mean it's plausible. Epidemiology (Cambridge, Mass). 2014;25(1):4-6.

5. Shrier I, Platt RW. Reducing bias through directed acyclic graphs. BMC Med Res Methodol. 2008;8:70-.

6. Pearce N, Lawlor DA. Causal inference—so much more than statistics. Int J Epidemiol. 2017;45(6):1895-903.

7. Blaine B. Does depression cause obesity?: A meta-analysis of longitudinal studies of depression and weight control. Journal of health psychology. 2008;13(8):1190-7.

8. Tyrrell J, Mulugeta A, Wood AR, Zhou A, Beaumont RN, Tuke MA, et al. Using genetics to understand the causal influence of higher BMI on depression. Int J Epidemiol. 2018;48(3):834-48.

9. Mannan M, Mamun A, Doi S, Clavarino A. Is there a bi-directional relationship between depression and obesity among adult men and women? Systematic review and bias-adjusted meta analysis. Asian journal of psychiatry. 2016;21:51-66.
